# Delayed rib plating and lung herniation repair in a traumatic chest injury after thoracotomy: A case report

**DOI:** 10.1016/j.ijscr.2024.109423

**Published:** 2024-02-27

**Authors:** Kella L. Vangsness, Janelle Lopez, Lauren Van Sant, Thomas Duncan, Graal Diaz

**Affiliations:** a Community Memorial Healthcare System, Ventura, CA 93003, United States of America; b Ventura County Medical Center, Ventura, CA 93003, United States of America

**Keywords:** Rib plating, Lung herniation, Traumatic chest injury, Thoracotomy, Vancomycin

## Abstract

**Introduction and importance:**

Lung herniation following trauma is a rare occurrence, and consensus on optimal surgical repair techniques remains limited. While small herniations may resolve without surgery, intervention becomes necessary in cases of unsuccessful non-operative management or concurrent rib fracture stabilization. Mesh application in repair poses a dilemma, often providing physical support but raising infection concerns, particularly in trauma scenarios with delayed closure. Surgical stabilization of rib fractures, employing hardware similar to orthopedic procedures, may necessitate prophylactic antibiotics, though empirical evidence supporting routine use is scant. Polytrauma patients often resort to delayed chest closure techniques during methodical surgical planning, but these carry potential consequences compared to immediate closure.

**Case presentation:**

Presented is a case involving a patient in a motorcycle collision sustaining multiple injuries, necessitating a massive transfusion protocol, multiple surgeries, including delayed chest closure, and eventual surgical rib fixation four days post-injury. During rib stabilization, exacerbation of traumatic lung herniation mandated mesh repair, prompting the cautious use of prophylactic vancomycin powder to mitigate infection risks.

**Discussion:**

A review of the literature revealed a scarcity of similar cases, particularly those involving lung herniation with delayed chest closure, the use of prophylactic antibiotics and mesh in polytrauma.

**Conclusion:**

This case underscores the lack of depth of comprehensive research guiding surgical decisions concerning lung herniation and the prophylactic use of vancomycin powder in trauma patients.

## Highlights

• Lung herniation following polytrauma
• Surgical stabilization of rib fractures
• Mesh application in repair
• Prophylactic antibiotics
• Delayed chest closure techniques

## 1 Introduction

Although rare, most lung herniations are reported in the setting of a traumatic injury [1] and found in the intercostal space as this area is only covered by external intercostal muscles. Current literature describes attempts to treat smaller, contained, and asymptomatic herniations non-operatively [[2], [3], [4]] - as many will heal spontaneously [5,6], while more significant and symptomatic cases will likely require surgical intervention [1]. There are multiple approaches to surgical interventions for lung herniation. These include but are not limited to repairing the defect primarily, surgical stabilization of rib fractures (SSRF) or sternal fractures (SSSF) if warranted, or placing a mesh [1,2,6].

The process of SSRF restores alignment through open reduction and internal fixation (ORIF), usually using titanium plates and screws^7,^ and is carried out when there is a physiologic or anatomic compromise to the area, such as flail chest or lung herniation. The routine prophylactic use of antibiotics in rib stabilization with internal hardware in the trauma setting is inconclusive as there is insufficient evidence available to provide recommendations for any patients with comorbidities such as sepsis, pneumonia, or empyema, or when performing SSRF or SSSF with an open or contaminated chest [7]. However, one study from the pediatric orthopedic literature demonstrated that vancomycin had fewer postoperative infections with only one microbe isolated compared to the control [8].

Primary chest closures have improved outcomes, and fewer interventions are required during hospital stays compared to secondary closures [10,11]. Secondary or delayed chest closure is often used for life-threatening situations to stabilize the patient first, such as polytrauma [12]. There are multiple methods described for delayed closure, including the use of negative pressure wound therapy, packing the chest with gauze, placement of chest tubes, or application of synthetic materials to cover the wound [[10], [11], [12]].

There are no reported cases or literature pertaining to the management of polytrauma patients who sustained multiple rib fractures, costochondral separation, delayed chest closure, and lung herniation repair with mesh. We present a unique case of delayed chest closure with lung herniation noted during closure after SSRF and SSSF, with the use of biologic bovine pericardium mesh and vancomycin powder in a polytrauma patient.

## 2 Case presentation

**Fig. 1:**
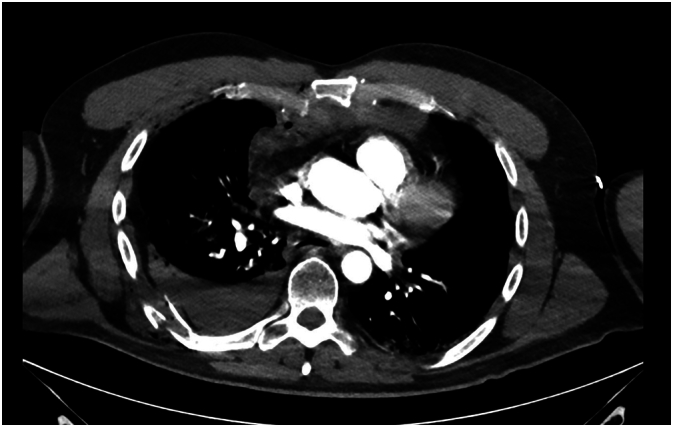
Computed tomography highlighting the costochondral separation of the right fourth intercostal space and right hemothorax.

**Fig. 2:**
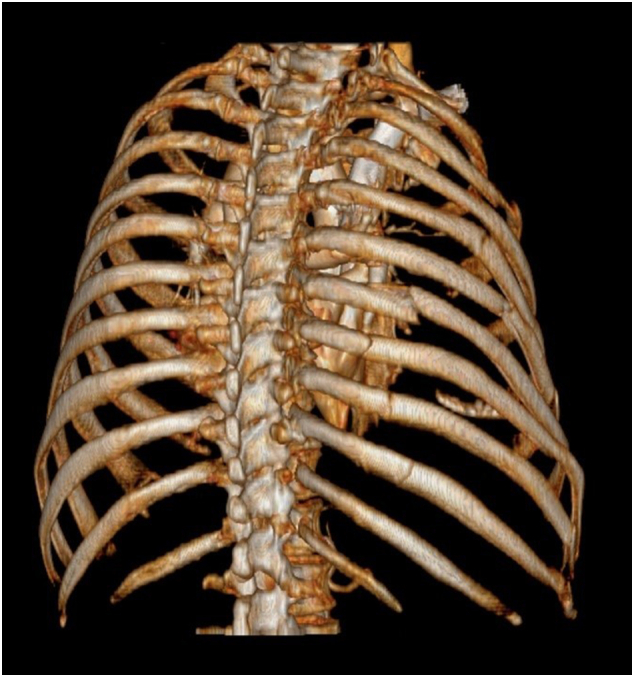
3D Computed tomography rendering demonstrating displaced posterior fractures of ribs five, seven to ten; nondisplaced posterior fracture of sixth rib.

**Fig. 3:**
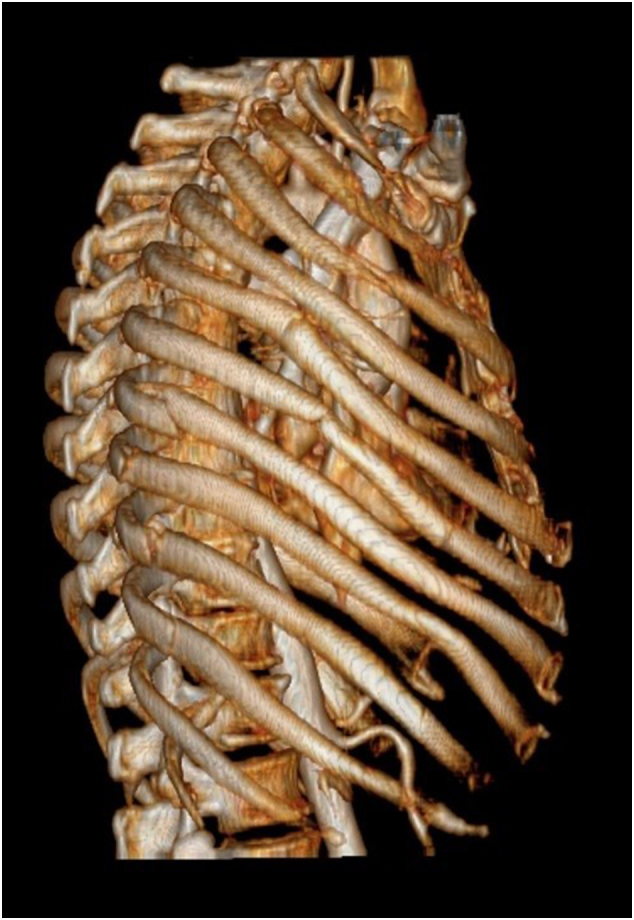
3D Computed tomography rendering demonstrating laterally displaced fractures of ribs five and six.

This is a 54-year-old male who was brought to the hospital as a trauma patient (Tier 2 activation) with a Glasgow Coma Scale (GCS) of fourteen after being found in a ditch from a motorcycle collision. He had a past medical history of tobacco, methamphetamine, and alcohol use. His urine drug screen was negative, and the alcohol screen was above the legal limit of intoxication (i.e.,>0.08). Computed tomography (CT) imaging showed a grade 3 blunt aortic injury, mild mesenteric injury, right adrenal gland hematoma, fractures of the right scapula, left clavicle, open book pelvic fracture, and multiple lumbar transverse process fractures. There was bleeding along the right sacroiliac joint extending into the pelvis. Injuries to his thorax included costochondral separation of right ribs one to five (Fig. 1), displaced posterior fractures of right ribs five and seven to ten, a nondisplaced posterior fracture of right rib six (Fig. 2), laterally displaced fractures of ribs five and six (Fig. 3), bilateral pulmonary contusions, and a right hemothorax. Upon return to the trauma bay from the CT scanner, he became hemodynamically unstable and was upgraded to a Tier 1 trauma activation (highest activation level). A right thoracostomy tube and pelvic binder were placed. He was intubated, and an arterial line was placed. A massive transfusion protocol was activated, and he was taken emergently to the hybrid Operating Room.

He underwent an exploratory laparotomy with colonic serosal tear repair, preperitoneal pelvic and abdominal packing, and temporary abdominal closure. Vascular surgery performed a thoracic endovascular aortic repair (TEVAR), and interventional radiology (IR) performed bilateral internal iliac artery embolizations. Despite these interventions, the patient remained hemodynamically unstable. Given that the right chest tube had >1.5 l output of sanguineous output, the decision to perform a right thoracotomy and place a left thoracostomy tube was made. No major hemorrhage was identified, the right chest was packed and temporarily closed, and the patient was taken to the intensive care unit (ICU).

Throughout the night, he remained hemodynamically unstable with continuous sanguineous output from the right chest tube. At this point, he had received twenty-four units of packed red blood cells (PRBC), twenty-four units of fresh frozen plasma (FFP), three units of cryoprecipitate (CRYO), and three rounds of plateletpheresis (PLT). The decision was made to return to the operating room (OR) for chest exploration. An intercostal artery transection was identified, likely due to further resuscitation and resolution of traumatic vasospasm, and hemorrhage control was obtained with ligation. He received a total of thirty-nine units of PRBC, thirty-six units of FFP, three units of CRYO, six rounds of PLT, and two units of whole blood (WB).

Post-operation day (POD) two, he returned to the OR for a re-exploration of his abdomen and thorax, and three transverse colon serosal injuries were repaired. A right lower lung pleural tear was identified and resected. The abdomen was closed, and his chest was again temporarily closed with the replacement of the thoracostomy tube and a plan for SSRF and SSSF the following day.

POD three, he underwent a difficult pelvic external fixation with sacroiliac screw placement and received an intraoperative transfusion of two units of PRBC, one unit of plasma, and one unit of PLT. The pelvic external fixation was to remain for six weeks with followup X-rays.

**Fig. 4:**
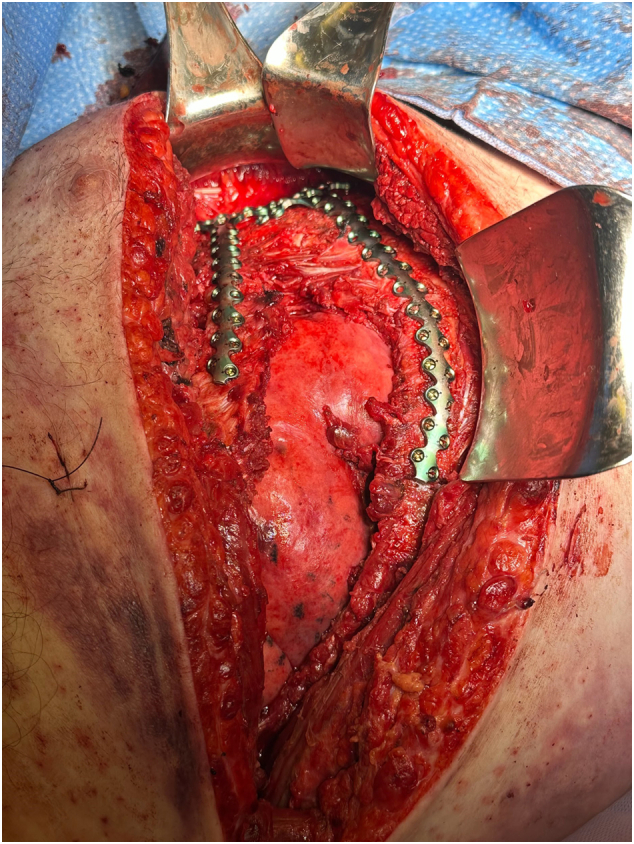
Surgical stabilization of rib fractures of the right anterior fourth and fifth ribs with sternal fixation.

**Fig. 5:**
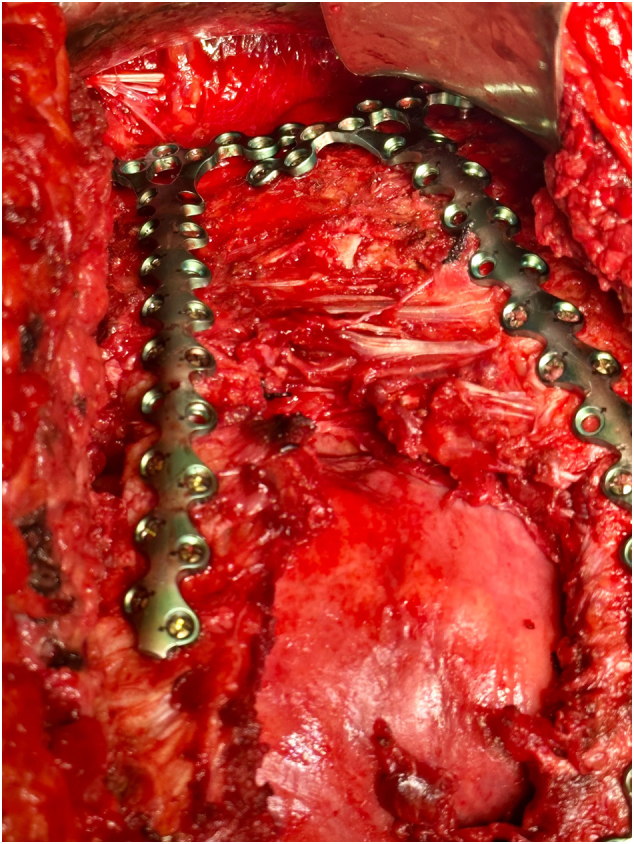
Highlights offset the placement of the plates on the sternum. To note, there is the anterior aspect of the fourth intercostal space is widened and where the biologic mesh was placed.

POD four from his original thoracotomy, he was taken back to the OR for definitive chest closure and rib fixation. The chest wall soft tissues were noted to have an area of fibrinous exudate, which was excised and sent for culture. A unicortical SSRF of the right posterolateral fifth and sixth ribs was performed. A unicortical SSRF of the right anterior fourth and fifth ribs with sternal fixation (Fig. 4) was performed to address the severe displacement of the costochondral junction. The thoracostomy tube was replaced. The fourth intercostal space was reapproximated with #1 vicryl. There was noted to be difficulty approximating the anterior aspect sufficiently (Fig. 4, Fig. 5), and the lung parenchyma was noted to be herniating at this location with every inhalation. The decision to place a mesh was made. Due to the nature of the chest wall being opened for four days (and limited mesh options), a bovine pericardium biologic mesh was sutured in place to the anterior wall of the pleural cavity with a 2-0 polydioxanone suture (PDS) suture in a “parachute” fashion. Vancomycin powder was placed in the area, and the muscle was reapproximated. A subcutaneous drain was inserted medially along the incision in the soft tissues, and a wound vacuum was placed to augment skin closure.

**Fig. 6:**
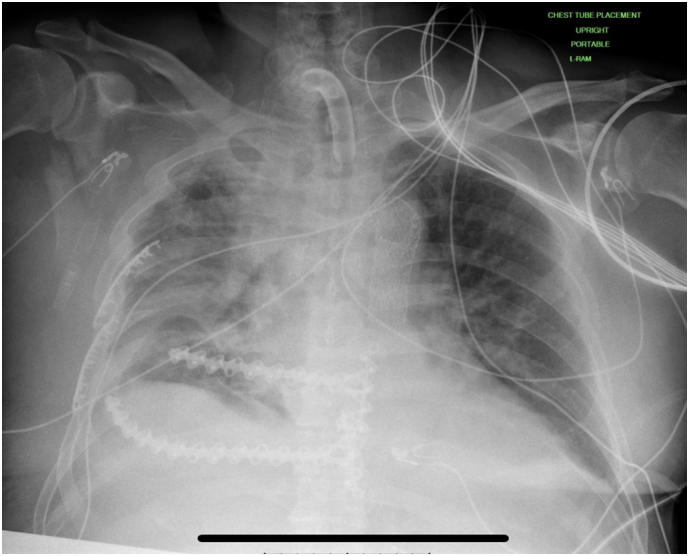
Chest X-ray demonstrating the surgical stabilization of rib fractures, once again showing the offset placement along the sternum. Noted is the stent placement from the thoracic endovascular aortic repair and the tracheostomy.

POD eight, he underwent a tracheostomy and received a percutaneous endoscopic gastrostomy (PEG) tube (Fig. 6). A bronchoalveolar lavage grew *Pseudomonas Aeruginosa* and Klebsiella Pneumonie, and he was started on appropriate antibiotics. The intraoperative chest wall exudate grew Bacillus, not Anthracis, and he was started on intravenous vancomycin. Progressing well, he stepped down from the ICU on POD sixteen. His thoracotomy tubes were removed. Eventually, the wound vacuum was removed, and a delayed primary closure of the thoracotomy incision was performed. POD twenty-six, his tracheostomy was decannulated, and he was started on an oral diet. POD thirty-two, he was declared to be as weight-bearing as possible and was transferred to a skilled nursing facility.

POD forty-nine, the pelvic external fixation apparatus was removed, and he was cleared to bear weight as tolerated. POD fifty-eight, he was seen at the clinic for removal of the PEG tube with no issues. Two weeks following the external fixation removal, he was seen in the orthopedic clinic and found to be improving significantly, ambulating with the assistance of a cane and beginning physical therapy.

## 3 Discussion

Lung herniation is either anatomic (cervical, intercostal, diaphragmatic) or etiological (congenital, acquired) with acquired mechanisms often induced by trauma and seen on initial presenting imaging [3,5]. The lung herniation in our case report was noted upon chest closure and not on original imaging. Etiologies of lung herniation in our case were most likely from the four-day duration the chest remained opened due to higher-priority injuries being addressed, the lack of intercostal muscle present at the site of lung herniation, and possibly the multiple ribs and costochondral separations that needed to be fixated.

Small, asymptomatic lung herniations are often treated non-operatively. In contrast, large, symptomatic lung hernias often require surgical management [6]. Most surgical interventions are performed after failure of non-operative management [[2], [3], [4],^12^]. Surgical management varies in terms of technique, use of mesh, and use of prophylactic antibiotics. SSRF has been utilized alone for lung herniation repair [4]. Rib fixation varies with the approach of muscle sparing versus non-muscle sparing [12,13], utilization of a monofilament suture in an interrupted manner [14], or ORIF with implanted hardware [1,5]. According to Fatmir, the indications for rib fixation include true flail chest, severely displaced rib fractures of three or more, and refractory pain or respiratory compromise [15]. In our case, in order to re-construct the chest wall, we performed fixation of the multiple severely displaced rib fractures and costochondral separations. In one case report, the costochondral separation was repaired with wire fixation [12]. We ensured the hardware along the sternum was offset during placement.

A variety of cases reported additional lung herniation repair techniques such as lung hernia sac excision [12], wedge resection of persistent herniated lung [3], partial rib resection [5], and mesh placement. Mesh noted in these reports consisted mainly of synthetic polytetrafluoroethylene mesh [3,5,12], and in some cases, due to increased concern of infection or desire for less chest wall restriction, soft biologic or bioprosthetic mesh was utilized [6,13]. Most surgical repairs utilized chest tubes [3,5.^13^]. We chose to use a biologic mesh due to concerns of increased risk of infection as chest closure was delayed for more than forty-eight hours. We chose to place chest tubes with every temporary thoracic wound closure application and at definitive chest closure.

The Surgical Infection Society's Therapeutics and Guidelines Committee and Chest Wall Injury Society's Publication Committee aimed to develop antibiotic recommendations for SSRF use. In their literature review, there was insufficient evidence available to provide recommendations for any patients with comorbidities such as sepsis, pneumonia, empyema, SSRF, or SSSF with open or contaminated chest [7]. The decision to use antibiotics prophylactically is not uncommon in the setting of open fractures, prolonged ICU stays, or in the setting of implanting hardware, particularly in the setting of increased infection possibilities such as a traumatic injury. Vancomycin powder was utilized in a prospective rib-based distraction surgery study. This study found lower infection rates and isolation of one microbe with vancomycin use compared to the control, which had multiple microbes on infection. There was little systemic penetration of the drug, no effect on creatinine, and no side effects. However, this patient population was not trauma-based nor specific for SSRF [8]. In a study of delayed chest closures for lung transplants, surgeons utilized prophylactic antibiotics of vancomycin and pathogen-specific antimicrobials. They found no statistically significant difference in infection rates at thirty days compared to the matched cohort [10].

The decision to place vancomycin powder was based on the potential risk of surgical infection with implant use in the setting of delayed chest wall closure; as infection with delayed thoracotomy closure is rare, but comes with high morbidity [16]. Vancomycin is commonly utilized in our facility as a prophylactic antibiotic against *Staphylococcus aureus*, which is shown as the most common pathogen in fracture related infection [[16], [17], [18]]. Our facility does have other antibiotics powders/pastes to cover other pathogens if indicated. Our patient did not experience surgical site infection nor infection of the implanted, although the intraoperative chest wall exudates growing Bacillus, not Anthracis. Of note, upon the result of microbe growth, he was started on intravenous vancomycin.

In the setting of polytrauma, our patient required multiple days of temporary chest closure. Examining existing literature on primary versus delayed chest closure following lung transplantation, primary closures had increased survival rates at day thirty and five years post-operatively [9], while delayed chest closures had an increased risk of surgical site infections, transfusions, intubations, severe primary graft dysfunction, worsened pulmonary function tests, prolonged hospital stays, and a higher risk of primary graft dysfunction [9,10]. Delayed techniques included skin closures and a superficial supportive device directly sutured in or held in place or using negative pressure wound therapy [9,10]. Specifically, these include a latex membrane such as Esmark or negative pressure wound therapy with commercially available devices [19] or the “Bogota Bag” technique utilizing three-liter saline bags with dressings and nasogastric tubes hooked to suction or chest tubes [10]. In a study of ICU patients with life-threatening thoracic injuries who underwent surgery, 5.6 % received a temporary closure for a mean delay of 3.3 days [11]. Techniques included chest gauze packing, a silastic sheet with or without packing, and a skin-only closure. We utilized various temporary thoracic closure techniques, including a “Bogota Bag,” packing with a chest tube, and Esmark stapled to the wound edges.

## 4 Conclusion

This case provides a unique technical and antibiotic management option for delayed chest wall closure and repair of lung herniation after injury. It highlights the need for more depth and consistency of research on this topic.

The SCARE checklist was utilized to write this care report [20]. A copy of the consent is available upon request. There was no funding for this study.

## Consent

Written informed consent was obtained from the patient for publication and any accompanying images. A copy of the written consent is available for review by the Editor-in-Chief of this journal on request.

## Ethical approval

The Ventura County IRB committee has given an exemption to this case report as ethics clearance was not necessary, there was no deviation from the standard ethical practices.

## Funding

No source of funding.

## Author contribution

Kella L. Vangsness: Writing original draft, visualization: Janelle Lopez, D.O., conceptualization, validation, investigation; Lauren Van Sant, D.O.: conceptualization, validation, investigation, Writing original draft; Thomas Duncan, Graal Diaz: Writing review & editing.

## Guarantor

Janelle Lopez DO.

## Research registration number

N/A.

## Conflict of interest statement

Nothing to declare.
